# The hidden burden of melioidosis in Nepal: a paradigm for the urgent need to implement a simple laboratory algorithm to detect *Burkholderia pseudomallei* in low-resource endemic areas

**DOI:** 10.1016/j.ijregi.2024.100377

**Published:** 2024-05-04

**Authors:** Suraj Bhattarai, Isabel Klugherz, Chiranjay Mukhopadhyay, Ivo Steinmetz

**Affiliations:** 1 Global Health Research & Medical Interventions for Development (GLOHMED), Lalitpur, Nepal; 2 Department of Clinical Research, London School of Hygiene and Tropical Medicine, London, UK; 3 Diagnostic & Research Institute of Hygiene, Microbiology and Environmental Medicine, Medical University of Graz, Graz, Austria; 4 Department of Microbiology, Kasturba Medical College, Manipal Academy of Higher Education, Manipal, India; 5 Center for Emerging and Tropical Diseases, Manipal Academy of Higher Education, Manipal, India

**Keywords:** Melioidosis, Burkholderia pseudomallei, Fever of unknown origin, POC tests, South Asia, Nepal

## Abstract

Melioidosis, an emerging infectious disease caused by the Gram-negative bacillus *Burkholderia pseudomallei*, is massively underdiagnosed in many low- and middle-income countries. The disease is clinically extremely variable, has a high case fatality rate, and is assumed to be highly endemic in South Asian countries, including Nepal. The reasons for underdiagnosis include the lack of awareness among clinicians and laboratory staff and limited microbiological capacities. Because costly laboratory equipment and consumables are likely to remain a significant challenge in many melioidosis-endemic countries in the near future, it will be necessary to make optimum use of available tools and promote their stringent implementation. Therefore, we suggest that health facilities in resource-poor countries, such as Nepal, introduce a simple and low-cost diagnostic laboratory algorithm for the identification of *B. pseudomallei* cultures. This screening algorithm should be applied specifically to samples from patients with fever of unknown origin and risk factors for melioidosis, such as diabetes. In addition, there could also be a role of low-cost, novel, promising serological point-of-care tests, which are currently under research and development.

## Highlights

• Melioidosis is predicted to be endemic in Nepal; however, there is a huge data gap.
• It is massively underdiagnosed due to the lack of awareness and diagnostic capacities.
• Simple diagnostic algorithms can help detect cases in resource-poor settings.

## Introduction

Melioidosis is an emerging infectious disease affecting humans and animals and is caused by *Burkholderia pseudomallei*, a Gram-negative bacillus present in the soil and surface water of tropical and subtropical regions [1]. There is growing evidence that the disease is massively underdiagnosed in many low- and middle-income countries. It is suggested that around 44% of the predicted total 165,000 annual cases worldwide occur in South Asian countries, including Nepal [2]. Owing to occupational exposure, rural farming populations are at a particular risk of melioidosis, especially during the rainy season. Other risk factors include aging and comorbidities, such as diabetes mellitus, alcoholism, and chronic kidney and lung disease [1]. Diabetes is, by far, the most important among those and present in up to 70% of cases [3]. Infected individuals might present with an extremely wide clinical spectrum, ranging from undiagnosed fever and localized infections in soft tissues and bones to severe pneumonia and septicemia, with case fatality rates from under 10% to 40% and higher [1]. Early diagnosis is essential because *B. pseudomallei* is inherently multiresistant to many commonly used empiric drugs. Once melioidosis is diagnosed, specific treatment can reduce mortality significantly [4]. No vaccine has been developed yet. Surprisingly, melioidosis is not on the World Health Organization list of neglected diseases, although the estimated disability-adjusted life years are much higher than many of the neglected tropical diseases on the World Health Organization list, such as dengue, schistosomiasis, or intestinal nematodes [5].

## Lack of awareness and limitations in the diagnosis of melioidosis in low-resource settings

There are multiple reasons for the under-recognition of melioidosis in many endemic countries such as Nepal. A positive *B. pseudomallei* culture from blood or any other clinical sample is the current diagnostic gold standard. However, this represents a serious hurdle; first, because of limited microbiological capacities and because the culture has a low sensitivity of around 60% [6]. In addition, owing to a lack of awareness, *B. pseudomallei* might not be recognized as such because this pathogen shares features with other environmental bacteria and, therefore, might be discarded as contaminant. Misidentification even in well-equipped laboratories can occur with commercial biochemical test systems or when commercial mass spectrometry methods with incomplete reference spectra in their databases are used [1]. However, in general, well-established biochemical, mass spectrometry and molecular polymerase chain reaction methods exist, which can reliably identify *B. pseudomallei* cultures. A major hurdle is that these methods are costly and may require expensive equipment and it is not foreseeable that those tools will be available in smaller laboratories in resource-constrained endemic areas in the near future. The same applies to the direct polymerase chain reaction detection of *B. pseudomallei* from clinical samples and currently available point-of-care (POC) antigen tests for the direct detection of *B. pseudomallei* lack sensitivity [7].

## Melioidosis cases can be detected through a low-threshold microbiological laboratory algorithm based on widely available standard tests

**Figure 1:**
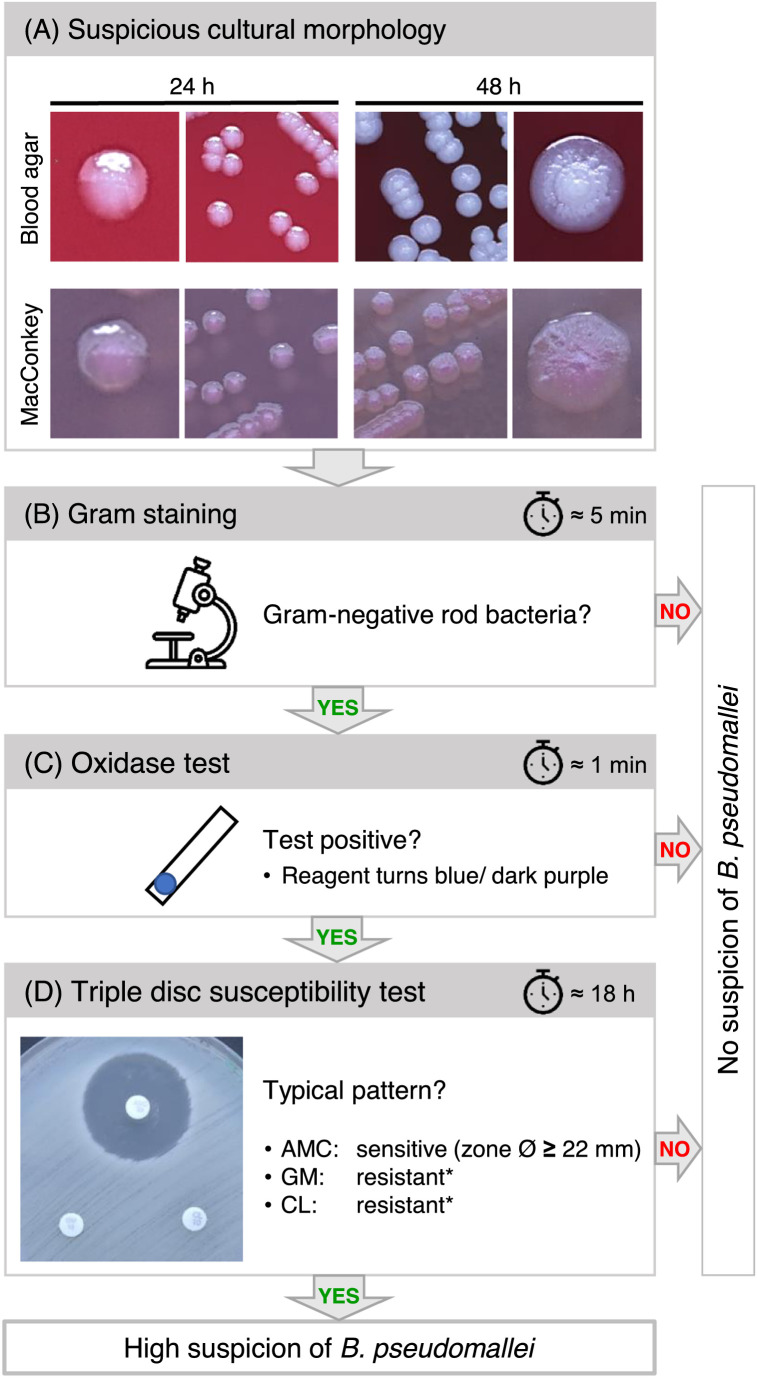
Simple microbiological algorithm for the presumptive identification of *B. pseudomallei* culture isolates from clinical samples. (a) Exemplary typical colony morphologies are shown on blood and MacConkey agar after the indicated incubation times at 37 °C. Depending on the agar medium, colonies can be creamy with a metallic sheen. After 48 h of incubation, colonies might appear drier and typically wrinkled, which can become more pronounced after longer incubation. Although *B. pseudomallei* is a lactose non-fermenting bacterium, colonies do not need to be colorless but can become pink after ≥48 h of incubation on MacConkey agar. (b), (c) The hands-on times for bacterial Gram staining and cytochrome c oxidase testing (e.g. oxidase reagent impregnated test strip) are indicated next to the clock symbols. (d) Susceptibility testing is performed according to EUCAST guidelines [20]. McFarland turbidity standards can be used to adjust the bacterial suspensions for the disc diffusion tests without a photometer.

AMC, amoxicillin/clavulanic acid (20/10 µg disc); CL, colistin (10 µg); GM, gentamicin (10 µg); h, hour; min, minute. *No break points are available for GM and CL: resistant isolates grow close to the antibiotic disc.

This worrying diagnostic constraint described above can be overcome by testing suspicious isolates obtained during routine procedures for a very characteristic inherent antibiotic resistance pattern of *B. pseudomallei.* This pathogen is a Gram-negative, cytochrome c oxidase-positive bacterium and resistant to colistin and gentamycin but susceptible to amoxicillin-clavulanic acid. This unusual antibiotic resistance pattern in the category of non-fermenting bacteria can be tested by widely available disc diffusion antibiotic susceptibility tests, which are also available in resource-limited microbiology laboratories. Figure 1 depicts the screening algorithm of suspicious cultures using Gram staining, cytochrome c oxidase testing, and antibiotic testing using a triple-disk diffusion susceptibility test [8].

This simple three-antibiotic disk test on Gram-negative, oxidase-positive rod bacteria is highly specific when applied to bacterial cultures isolated from clinical samples [8]. Studies from Vietnam have shown that the introduction of this algorithm enabled laboratories to identify *B. pseudomallei* in parts of the country where the disease has not previously been recognized [8], [9]. In a study from north-central Vietnam, all 94 suspected strains detected via the triple-disk test from 76 blood cultures and 18 other samples could be confirmed by molecular methods [8]. In a study from south Vietnam, 43 of 44 strains detected through the triple-disk test were confirmed as *B. pseudomallei*. Those isolates were derived from nine blood cultures and 34 other samples [9]. This simple screening approach is applicable with culture media in routine use by local laboratories. The ability to identify an important medical problem using a simple procedure with a high positive predictive value is particularly motivating for small health care facilities in remote areas. Subsequently, definitive confirmation of *B. pseudomallei* can be obtained in, for example, reference centers. If cases are detected, laboratories can then decide to introduce additional media containing selective agents, which might further increase the detection rate [10]. Most importantly, positive triple-disk tests can lead to adequate melioidosis therapy being carried out in resource-constrained regions. However, one must still consider that the successful and sustainable application of even such a simple algorithm requires adherence to the basic principles of quality control in the microbiology laboratory (e.g. verification of tests using positive and negative controls, etc.).

Because the search for *B. pseudomallei* in environmental samples is also an important measure to identify risk areas for melioidosis, it is necessary to mention that the high specificity of the triple-disk test is valid for clinical samples but may not apply for environmental samples. The background is that other related environmental species within the *B. pseudomallei* complex exist [11], which might show the same susceptibility pattern in the triple-disk test but have either no or only very limited virulence (e.g. *B. thailandensis*) and are, therefore, only very rarely isolated from humans. *B pseudomallei* gentamicin-sensitive strains do exist; however, apart from a local clade present in Malaysia, they are only rarely isolated. An exceedingly low prevalence (<0.1%) applies for amoxicillin/clavulanic acid–resistant *B pseudomallei* strains from primary melioidosis, whereas occasionally acquired amoxicillin/clavulanic acid resistance can be detected in relapsed melioidosis [11].

## Reported cases from Nepal are likely to be a very tiny tip of the iceberg

There have only been a few melioidosis cases reported so far from Nepal. A case of pulmonary melioidosis was published in 2005 and labeled as imported because the patient had returned from Malaysia, a known endemic area, 1 month before the onset of symptoms [12]. Two more recent cases reported in 2019 had a travel history to Malaysia; however, both patients were involved in farming activities in Nepal before the onset of symptoms and the time between the return from Malaysia and the onset of symptoms was several years [13], making it most probable that these were “indigenous cases.” Both patients had diabetes with multiple sites of infection, including pulmonary involvement. Beyond that, a fatal case of cerebral melioidosis of a young Nepalese serving soldier with no reported travel history was published in 2019 [14]. Finally, a primary cutaneous melioidosis acquired in Nepal in a Dutch patient and diagnosed in the Netherlands was reported in 2021 [15].

The following three facts support our claim that melioidosis could be widely present in Nepal. First, almost two-thirds of the total population of Nepal are engaged in agriculture, and approximately 28 million people live in the southern plains (Tarai) and the hill region, which are the areas with environmental suitability for *B. pseudomallei*. Second, the environmental conditions in southern Nepal are very similar to the Indian States along Nepal's southern line, where melioidosis cases have been reported in four States (West Bengal, Jharkhand, Uttar Pradesh, and Bihar; cases are being collected by co-author: CM) [16]. Third, there is a huge burden of diabetes in Nepal, with a prevalence of 10% and 13% among those aged 40-59 and >60 years, respectively [17].

## A potential new role for serology in the diagnosis of melioidosis through recent test developments

Apart from the previously mentioned limited sensitivity of *B. pseudomallei* detection depending on bacterial cultures from patient samples, the detection is also made difficult by the necessity to collect adequate and often invasive samples. Because most patients with severe disease have already developed antibodies when presenting with symptoms, this could be potentially avoided by serological POC methods that use very low blood volumes, for example, obtained from a finger prick. Such an approach would even be feasible for small health care centers without laboratory access. In the past, antibody detection using crude antigen preparations suffered from a lack of standardization, poor sensitivity in the diagnosis of acute infections, and insufficient specificity due to high background seropositivity. In this context, recent developments in the identification of *B. pseudomallei*-specific antigens and their use in serological POC assays to diagnose melioidosis are encouraging [18,19].

## Conclusion

We underscore in this article the urgent need for training and research to determine the real burden of melioidosis in Nepal. The introduction of a simple and low-cost diagnostic laboratory algorithm for the identification of *B. pseudomallei* targeted at samples from patients with fever of unknown origin and risk factors for melioidosis is of utmost importance. It is reasonable to focus primary efforts on blood cultures because of the high rate of bacteremic melioidosis. Local workshops and lectures, including basic principles of quality assurance, will be crucial in bringing this knowledge to remote areas. Future studies will show whether novel promising serological POC tests will be an attractive diagnostic addition with a clinical impact in resource-constrained settings.

## Declarations of competing interest

The authors have no competing interests to declare.
